# Optimizing adjuvant treatment options for patients with glioblastoma

**DOI:** 10.3389/fneur.2024.1326591

**Published:** 2024-02-21

**Authors:** Enzhao Zhu, Jiayi Wang, Weizhong Shi, Qi Jing, Pu Ai, Dan Shan, Zisheng Ai

**Affiliations:** ^1^School of Medicine, Tongji University, Shanghai, China; ^2^Shanghai Hospital Development Center, Shanghai, China; ^3^Department of Anesthesiology and Perioperative Medicine, Shanghai Fourth People’s Hospital, School of Medicine, Tongji University, Shanghai, China; ^4^Faculty of Health and Medicine, Lancaster University, Lancaster, United Kingdom; ^5^Department of Medical Statistics, School of Medicine, Tongji University, Shanghai, China; ^6^Clinical Research Center for Mental Disorders, Chinese-German Institute of Mental Health, Shanghai Pudong New Area Mental Health Center, School of Medicine, Tongji University, Shanghai, China

**Keywords:** glioblastoma, radiotherapy, chemoradiotherapy, deep learning, machine learning

## Abstract

**Background:**

This study focused on minimizing the costs and toxic effects associated with unnecessary chemotherapy. We sought to optimize the adjuvant therapy strategy, choosing between radiotherapy (RT) and chemoradiotherapy (CRT), for patients based on their specific characteristics. This selection process utilized an innovative deep learning method.

**Methods:**

We trained six machine learning (ML) models to advise on the most suitable treatment for glioblastoma (GBM) patients. To assess the protective efficacy of these ML models, we employed various metrics: hazards ratio (HR), inverse probability treatment weighting (IPTW)-adjusted HR (HR^a^), the difference in restricted mean survival time (dRMST), and the number needed to treat (NNT).

**Results:**

The Balanced Individual Treatment Effect for Survival data (BITES) model emerged as the most effective, demonstrating significant protective benefits (HR: 0.53, 95% CI, 0.48–0.60; IPTW-adjusted HR: 0.65, 95% CI, 0.55–0.78; dRMST: 7.92, 95% CI, 7.81–8.15; NNT: 1.67, 95% CI, 1.24–2.41). Patients whose treatment aligned with BITES recommendations exhibited notably better survival rates compared to those who received different treatments, both before and after IPTW adjustment. In the CRT-recommended group, a significant survival advantage was observed when choosing CRT over RT (*p* < 0.001). However, this was not the case in the RT-recommended group (*p* = 0.06). Males, older patients, and those whose tumor invasion is confined to the ventricular system were more frequently advised to undergo RT.

**Conclusion:**

Our study suggests that BITES can effectively identify GBM patients likely to benefit from CRT. These ML models show promise in transforming the complex heterogeneity of real-world clinical practice into precise, personalized treatment recommendations.

## Introduction

Glioblastomas (GBM), the most prevalent and lethal malignant brain tumors in adults (1), have a dire 5 years survival rate of merely 6.8% (2). Despite extensive research, survival rates for central nervous system malignancies have not significantly improved, underscoring the need for enhanced therapeutic approaches (1, 3).

While promising therapies like monoclonal antibodies (4), immunotherapy (5), and oncolytic viruses are under investigation (6), their clinical efficacy requires further validation (7), and traditional treatments—surgical resection followed by radiotherapy (RT) or chemoradiotherapy (CRT)—prevail (8). RT, a mainstay in GBM management, aims to boost local control and overall survival and continues to be a critical treatment modality (9). CRT, which was shown in 2005 to increase 2 years median survival to 26.5% compared to RT alone’s 10.4% (10), has become a standard GBM treatment. However, adjuvant chemotherapy’s (CT) toxicities, such as nausea and myelosuppression, are notable, especially during adjuvant treatment (8), and its effectiveness varies among patients with differing features (11, 12). CRT’s associated toxicity may not be tolerable for elderly patients, rendering it more appropriate for fit individuals under 70 (10, 13). Consequently, optimizing adjuvant therapy based on patient characteristics to reduce treatment costs and toxicity is a critical concern.

The traditional method of addressing this involves stratifying GBM patients into subgroups based on their characteristics and conducting randomized controlled trials (RCTs) in each subgroup to evaluate RT versus CRT. However, RCTs are always time-consuming and costly, and thus difficult to recruit a large number of patients in real-world applications (14). Moreover, implementing RCTs may face ethical constraints, as it is very challenging to assign a sole RT treatment to patients, especially when existing evidence suggests that CRT prolongs patients’ survival, and when there is a lack of clear evidence regarding which features potentially affect the efficacy of conjoint CT treatment. Instead of RCTs, observational evidence, therefore, becomes an attractive alternative. Yet, determining whether a patient experiences improved survival when treated with CRT rather than RT poses certain challenges. This is primarily due to the fact that a patient cannot simultaneously receive both treatments, and confounding variables are prevalent in observational studies (15). Benefitting from advances in machine learning (ML) and statistical theories, we can use balanced representation-based (16), tree-based (17), and conditional average treatment effect (CATE)-based (18, 19) methods to counterfactually infer patients’ individual treatment effect (ITE) directly from observational data and thus attempt identify the relatively optimal treatment choice for specific individuals. With the development of deep learning (DL) and representation learning, novel techniques enable combining DL with survival models and learning balanced representations directly from the data to reason about unbiased counterfactual survival outcomes (20).

This study therefore used a novel DL model to analyze the ITE of GBM patients to infer potential survival improvements (e.g., survival time and survival probability) CRT could offer over RT for individual patients. The interpretations of the DL model are expected to yield features relevant to treatment selection and provide *a priori* evidence for subsequent prospective studies.

## Materials and methods

### Study design

This was a retrospective cohort study that used the state-of-the-art DL approach to counterfactually predict the ITE of patients with GBM to determine whether an individual is better suited to receive RT or CRT. All participants included in this study were selected from the Surveillance, Epidemiology, and End Results 18 (SEER 18) database, which tracks patients with cancer from 18 regions of the United States, and the population in SEER 18 represents approximately 27.8% of the US population (21). The patients and treatments included in this study therefore very closely resemble real-world distributions. This study followed the Strengthening the Reporting of Observational Studies in Epidemiology reporting guidelines (22).

**Figure 1:**
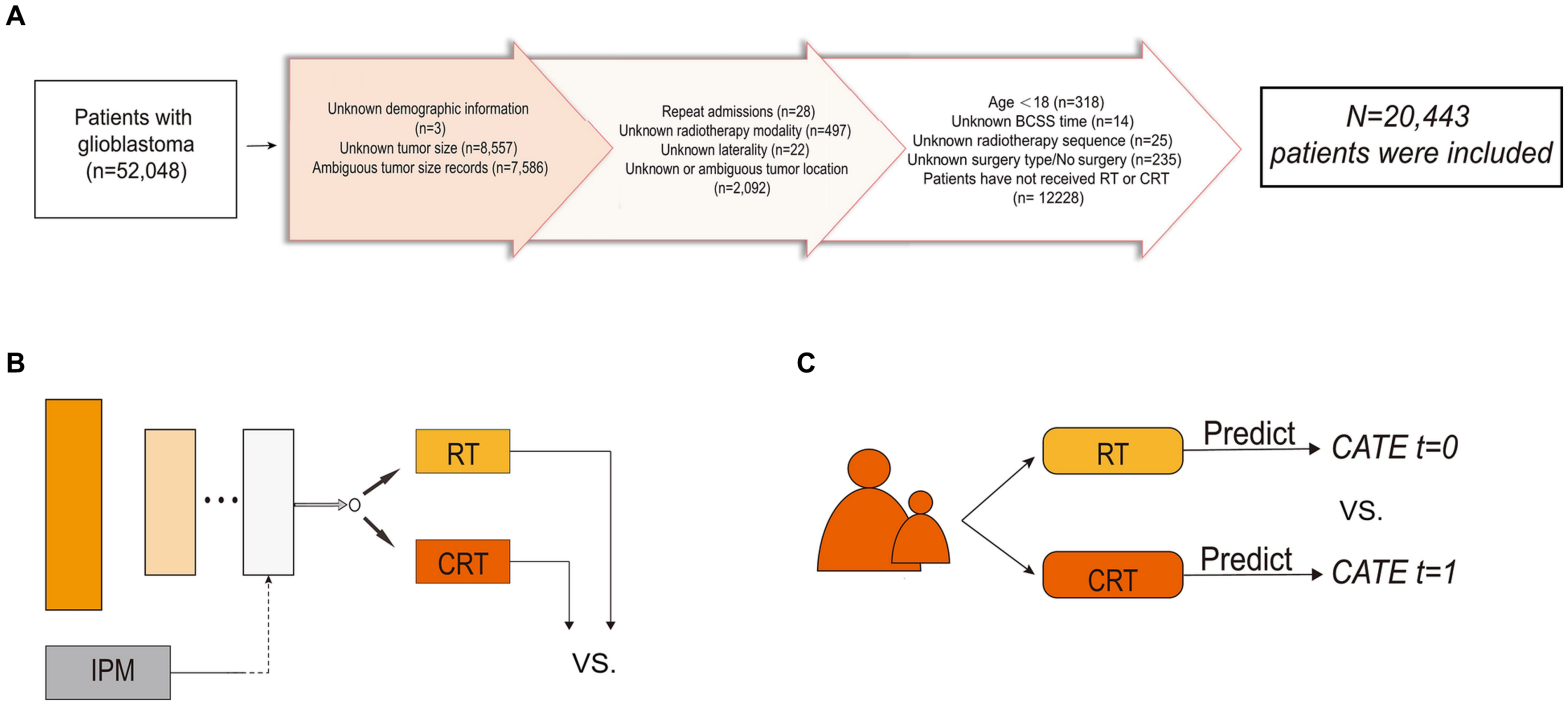
Flowchart of patient inclusion and schematic diagram of the model structure. **(A)** Flowchart of patient inclusion. **(B)** Schematic diagram of balanced individual treatment effect for survival data model. **(C)** Schematic diagram of T-learner. IPM, integral probability metrics; RT, radiotherapy; CRT, chemoradiotherapy; CATE, conditional average treatment effect.

The inclusion criteria were as follows: (1) patients diagnosed with GBM as primary cancer from 2005 to 2015, and (2) patients who received post-operative RT or CRT. The sequence of operation on CT is not provided by SEER; hence, no constraints are placed on its order. The exclusion criteria were as follows: (1) age < 18 years; (2) unknown tumor location, size, or laterality; (3) unknown whether the surgery had been performed or the surgery type; (4) unknown sequence of surgery and RT; (5) unknown survival time; (6) repeat admissions; (7) unknown patients’ demographic information; and (8) unknown RT modality. The comprehensive procedure for incorporating the study population is depicted in Figure 1A. We collected patients’ baseline demographic information (sex, age, marital status, living area, economic status, and reporting state), information related to the tumor (tumor size, primary location, laterality, extension, and metastasis), and treatment details [i.e., the extent of resection (EOR) and adjuvant treatments]. Tumor size was recorded at the time of diagnosis and referred to as tumor diameter. We defined the outcome of interest as brain cancer-specific survival (BCSS), which is the time interval between the diagnosis of GBM and the final death caused by the brain tumor.

### Machine learning algorithm

Unbalanced features between treatment groups in observational studies exist due to the presence of confounding factors and treatment selection bias (23). The CATE-based method, by splitting the entire group into homogenous subgroups, is a representative method to adjust for confounders and selection bias. Ideally, in each treatment arm, patients are similar under certain measurements over the covariates; therefore, the participants in the same subgroup can be viewed as samples under RCT. The two-learner (T-learner) trains an ML model in each of the two treatment populations separately. Each model represents a hypothesis of treatment during reasoning and yields the CATE. A schematic diagram of the T-learner is presented in Figure 1B.

T-learner excludes some confounding artifacts; however, it can still be affected by inconsistent predictive performance and biased treatment allocation (14). To address this issue, we utilized Balanced Individual Treatment Effect for Survival data (BITES) (20), one of the recently proposed DL models capable of making individual-level causal inferences, so as to predict each patient’s ITE and to make treatment recommendations for GBM patients (24). BITES combines both representation-based and CATE-based causal inference methods, therefore providing more unbiased ITE inferences. The network structure of the BITES is presented in Figure 1C. BITES contains a shared network used for feature extraction and distribution balancing and two risk networks that each represent a specific treatment population. Balancing the generating distributions of treatment groups has been proven to be effective for both covariate space (25) and latent representations (26). Thus, BITES uses integral probability metrics (IPM) to quantify and maximize the difference in probability measures between different treatment populations (27). At the same time, a similar structure to the T-learner was achieved by feeding the potential features of patients receiving different treatments into the corresponding risk networks. However, unlike the T-learner, which trains two different models, the BITES model is trained end-to-end.

Cox mixtures with heterogeneous effects (CMHE) is a recently proposed DL model that extends the Cox proportional hazards model (CPH) with the effect of confounders and treatment (28). The CPH assumes that individuals across the population have constant proportional hazards over time, which is a strong assumption. CMHE assumes that latent clusters with different risk groups exist, and the proportional hazards assumption holds within each latent cluster, called the conditional proportional hazards assumption. CMHE uses the stochastic expectation maximization algorithm to balance the generating distributions of risk groups (29). DeepSurv (30) is a semiparametric model that replaces the linear model of CPH with multilayer perceptron.

The training and inference of CPH, DeepSurv, survival tree (ST), and random survival forest (RSF) were in the same format as T-learner, while BITES and CMHE were used in the same way as presented in the original paper.

### Inference of individual treatment effect

For the ITE estimation, there are two possible treatments, RT and CRT, while only a single factual can be observed and the alternative situation is missing. Let the ITE of individual i be defined as ${ITE}_{i}=Y_{i}\left(X_{i}|\;\mathrm{do}\;1\right)-Y_{i}\left(X_{i}|\mathrm{do}\;0\right)$, where do1 indicates the situation in which a patient received CRT, do0 indicates the situation in which a patient received RT, and Y is the outcome. In time-to-event prediction, the outcome measurements vary (31, 32). We defined the outcome as the length of time that an individual patient’s mortality reached 50% from the beginning.

After comparing ITE, we can obtain individualized recommendations from the model. We further divided the patients into consistent (Consis.) and inconsistent (Inconsis.) groups based on whether the actual treatment they received was consistent with the model recommendations.

### Model training, validation, and interpretation

We allocated 80% of the overall patients as the training set for model development and the remaining 20% as the testing set, unseen from the models during the training process, for performance evaluation. For training, we utilized 3-fold cross-validation that trains on two-thirds of the training set and validates the remaining training set. We used decoupled weight decay regularization (33) to optimize the model parameters.

We calculated the concordance index (C-index) and integrated Brier score (IBS) as regular discrimination performance metrics. We used the hazard ratio (HR), the difference in restricted mean survival time (dRMST), and number needed to treat (NNT) to evaluate the recommendation effect. We also used inverse probability treatment weighting (IPTW)-adjusted HR (HR^a^), which was adjusted for all the covariates, to provide a more causal inference for the recommendation effect.

SurvSHAP(t) (34) is a recently proposed time-dependent explainability of any survival model prediction that is based on SHapley Additive exPlanations (SHAP) with solid theoretical foundations (35). SurvSHAP(t) satisfies the local accuracy property and accurately explains the predictions of the model in the form of a survival function, describing varying contributions across the entire range of times analyzed.

### Statistical analysis

R 4.1.3 and Python 3.8 were used for statistical analyses. Continuous variables are reported as the median and interquartile range (IQR), and categorical variables are presented as counts and percentages (%). Kaplan–Meier (K–M) curves were compared using the log-rank test. The chi-square test was used to compare the categorical variables. The NNT was defined as the restricted mean survival time (RMST) in the Consis. group divided by the dRMST between the Consis. and Inconsis. groups up to a chosen time of 5 years, which was proposed by Yang and Yin (36).

## Results

### Demographic and clinicopathological characteristics

A total of 20,443 patients with complete BCSS records were included in this study, with a median follow-up time of 12 (6–21) months and an overall BCSS mortality rate of 75.9% [95% confidence interval (CI): 75.3–76.5%]. The median age was 62 (54–70) years, and 40.6% of patients were male. Among the tumor-related variables, the sites with the highest incidence of tumors in the total population were the frontal [6,344 (31.0%)], temporal [6,146 (30.1%)], and parietal [3,596 (17.6%)]. All patients underwent surgery for primary cancer. The extent of resection can range from biopsy [3,984 (19.5%)] to subtotal resection (STR) [4,864 (23.8%)], gross total resection (GTR) [5,869 (28.7%)], and supratotal resection (SpTR) [5,726 (29.0%)].

The detailed baseline clinical characteristics of those who underwent RT and those who underwent CRT are presented in Table 1. Among them, 2,089 (10.2%) patients received RT, and 18,354 (89.8) patients received CRT. The mortality rate of BCSS in the RT group was significantly higher than that in the CRT group (80.4% vs. 75.4%, *p* < 0.001).

**Table 1:** Baseline demographic and tumor-related information.

	Adjuvant radiotherapy (*n* = 2,086)	Adjuvant chemoradiotherapy (*n* = 18,354)
Age, median (range), y	69 (58–76)	61 (53–69)
Tumor size, median (range), mm	45 (33–56)	45 (32–56)
Married	1,258 (60.2)	12,647 (68.9)
Urban	1,837 (87.9)	16,242 (88.5)
*Sex*		
Female	903 (43.2)	7,401 (40.3)
Male	1,186 (56.8)	10,953 (56.7)
*Race*		
White	1,851 (88.6)	16,460 (89.7)
Other	238 (11.4)	1,894 (10.3)
*Area of US*		
Midwest	1,257 (60.2)	11,880 (64.7)
East	417 (20.0)	3,173 (17.3)
South	403 (19.3)	3,141 (17.1)
Oversea	12 (0.6)	160 (0.9)
*Income (US dollar)*		
Lower than $55,000	553 (26.5)	4,878 (26.6)
Higher than $55,000	1,536 (73.5)	13,476 (73.4)
*Location*		
Frontal	618 (29.6)	5,726 (31.2)
Temporal	632 (30.3)	5,512 (30.0)
Parietal	336 (17.5)	3,230 (17.6)
Occipital	85 (4.1)	902 (4.9)
Cerebellum	21 (1.0)	125 (0.7)
Cerebrum	49 (2.3)	337 (1.8)
Brainstem	4 (0.2)	31 (0.2)
Ventricle	5 (0.2)	41 (0.2)
Overlapping	307 (14.7)	2,450 (13.3)
*Laterality*		
Left	842 (40.3)	8,005 (43.6)
Mid	248 (11.9)	1,459 (7.9)
Right	999 (47.8)	8,890 (48.4)
*Tumor extension*		
Confined	1,776 (85.1)	15,738 (85.7)
Ventricular system	56 (2.7)	487 (2.7)
Midline	254 (12.2)	2,129 (11.6)
*Metastasis*		
Yes	571 (27.3)	6,101 (33.2)
*Brain cancer-specific survival*		
Censored	409 (19.6)	4,510 (24.6)
Dead	1,680 (80.4)	13,844 (75.4)

### Model performance and treatment recommendation

The detailed model performance and treatment recommendation effect are presented in Table 2. In the RT group, CMHE had the highest C-index (0.63, 95% CI: 0.62–0.65), followed by CPH (0.60, 95% CI: 0.57–0.64) and RSF (0.58, 95% CI: 0.55–0.63). ST had the lowest C-index (0.53, 95% CI: 0.50–0.56). BITES did not achieve a high C-index (0.55, 95% CI: 0.51–0.58), but had the best IBS (0.05, 95% CI: 0.04–0.07), which indicates a better probabilistic fit for survival. CMHE ranked second (0.06, 95% CI: 0.04–0.07), and CPH ranked third (0.07, 95% CI, 0.06–0.09) for IBS. The IBS of the ST in the RT group was significantly worse than that in the other models (0.12, 95% CI: 0.10–0.14). In the CRT group, CPH had the best C-index (0.64, 95% CI: 0.63–0.65), followed by CMHE (0.63, 95% CI: 0.63–0.64), DeepSurv (0.63, 95% CI: 0.62–0.64), BITES (0.62, 95% CI: 0.60–0.63), and RSF (0.62, 95% CI: 0.61–0.63). There is no significant difference in the C-index of the above models. CMHE achieved the best IBS (0.08, 95% CI: 0.07–0.08), followed by BITES (0.08, 95% CI: 0.07–0.09) and CPH (0.08, 95% CI: 0.07–0.08). Both the IBS and C-index of ST in the CRT group were significantly worse than those in the other models (C-index: 0.54, 95% CI, 0.53–0.55; IBS: 0.15, 95% CI, 0.14–0.16).

**Table 2:** Model performance and recommendation effect.

Model	HR	HR^a^	dRMST	NNT	C-index^b^	IBS^b^	C-index^c^	IBS^c^
BITES	**0.53 (0.48–0.60)**	**0.65 (0.55–0.78)**	**7.92 (7.81–8.15)**	**1.67 (1.24–2.41)**	0.55 (0.51–0.58)	**0.05 (0.04–0.07)**	0.62 (0.60–0.63)	0.08 (0.07–0.09)
CMHE	0.55 (0.49–0.62)	0.69 (0.58–0.81)	7.39 (5.62–9.16)	1.85 (1.34–2.78)	**0.63 (0.62–0.65)**	0.06 (0.04–0.07)	0.63 (0.63–0.64)	**0.08 (0.07–0.08)**
DeepSurv	0.68 (0.62–0.75)	0.80 (0.68–0.93)	5.10 (4.98–5.26)	3.16 (2.28–4.85)	0.55 (0.52–0.58)	0.08 (0.06–0.10)	0.63 (0.62–0.64)	0.11 (0.10–0.11)
CPH	0.54 (0.48–0.61)	0.66 (0.56–0.79)	7.63 (7.50–7.81)	1.76 (1.28–2.65)	0.60 (0.57–0.64)	0.07 (0.06–0.09)	**0.64 (0.63–0.65)**	0.08 (0.07–0.08)
ST	0.94 (0.88–1.01)	0.96 (0.89–1.03)	0.83 (0.73–0.95)	23.51 (9.43–55.56)	0.53 (0.50–0.56)	0.12 (0.10–0.14)	0.54 (0.53–0.55)	0.15 (0.14–0.16)
RSF	0.58 (0.52–0.64)	0.66 (0.57–0.77)	7.13 (5.67–8.97)	1.97 (1.45–2.90)	0.58 (0.55–0.63)	0.08 (0.06–0.09)	0.62 (0.61–0.63)	0.09 (0.08–0.09)

BITES, balanced individual treatment effect for survival data; CMHE, Cox mixtures with heterogeneous effects; CPH, Cox proportional hazards model; ST, survival tree; RSF, random survival forest; HR, hazards ratio; HR^a^, inverse probability treatment weighting adjusted HR; dRMST, the difference in 5 years restricted mean survival time; NNT, number needed to treat; C-index, concordance index; IBS, integral Brier score; ^b^, the indicator of radiotherapy group; ^c^, the indicator of chemoradiotherapy group. Bolded values indicate the best performance of the model in this metric.

BITES referred 4,034 (98.7%) patients for CRT treatment and 55 (1.3%) for RT only; 450 (11.0%) patients were in the Inconsis. group. CMHE referred 18 (0.5%) patients for RT treatment, and 439 (10.7%) were in the Inconsis. group. DeepSurv referred 414 (10.1%) patients for RT treatment, while 723 (17.7%) patients were in the Inconsis. group. ST recommended 1,463 (35.8%) patients for RT treatment, and 2,467 (60.3%) were in Consis. group. CPH referred all patients for CRT treatment, and 425 (10.4%) of patients were in the Inconsis. group.

HR^a^ indicated the HR value adjusted for all covariates included in this study using IPTW. The protective effect of BITES is the strongest of all models both before and after the correction (HR: 0.53, 95% CI, 0.48–0.60; HR^a^: 0.65, 95% CI, 0.55–0.78; dRMST: 7.92, 95% CI, 7.81–8.15), followed by CPH (HR: 0.54, 95% CI: 0.48–0.61; HR^a^: 0.66, 95% CI, 0.56–0.79; dRMST: 7.63, 95% CI, 7.50–7.81), CMHE (HR: 0.55, 95% CI, 0.49–0.62; HR^a^: 0.69, 95% CI, 0.58–0.81; dRMST: 7.39, 95% CI, 5.62–9.16), RSF (HR: 0.58, 95% CI, 0.52–0.64; HR^a^: 0.66, 95% CI, 0.57–0.77; dRMST: 7.13, 95% CI, 5.67–8.97), and DeepSurv (HR: 0.68, 95% CI, 0.62–0.75; HR^a^: 0.80, 95% CI, 0.68–0.93; dRMST: 5.10, 95% CI, 4.98–5.26). The HR and HR^a^ of ST did not show statistically significant protective effects (HR: 0.94, 95% CI, 0.88–1.01; HR^a^: 0.96, 95% CI, 0.89–1.03), while the 5 years dRMST showed a slight protective effect (0.83, 95% CI: 0.73–0.95). The NNT measures the number of patients who need to change their treatment based on model recommendations to prevent BCSS events within 5 years. In the same trend, BITES had the best NNT (1.67, 95% CI: 1.24–2.41), which was significantly better than that of DeepSurv (3.16, 95% CI: 2.28–4.85) and ST (23.51, 95% CI: 9.43–55.56), followed by CPH (1.76, 95% CI: 1.28–2.65), CMHE (1.85, 95% CI: 1.34–2.78), and RSF (1.97, 95% CI: 1.45–2.90).

**Figure 2:**
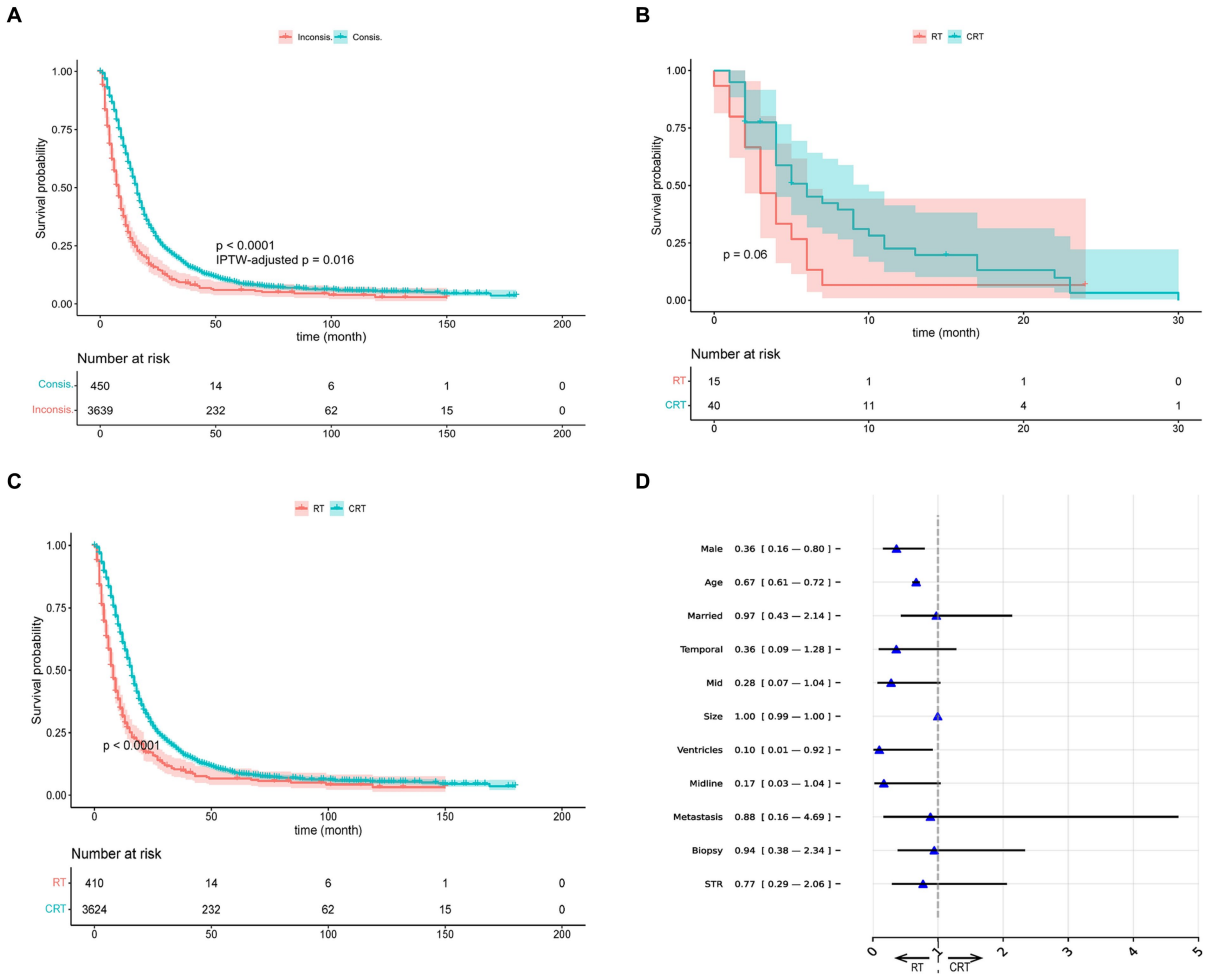
Visualizations of recommendation effects and behavior. **(A)** The K–M curves of Consis. and Inconsis. group. **(B)** The K–M curves of radiotherapy (RT) and chemotherapy (CRT) group in recommended RT group. **(C)** The K–M curves of radiotherapy (RT) and chemotherapy (CRT) group in recommended CRT group. **(D)** The odds ratio of BITES recommendation behavior. IPTW, inverse probability treatment weighting; STR, subtotal resection.

In addition, we presented the K–M curves (*p* < 0.001; IPTW-adjusted *p* = 0.016) of Consis. and Inconsis. groups of BITES in Figure 2A. We then divided the patients into recommended RT (RRT) and recommended CRT (RCRT) groups according to the recommendations of the model and evaluated the treatment effect of RT and CRT within each group. The K–M curves of the RT and CRT groups in the RRT group are presented in Figure 2B, in which CRT did not show a statistically significant survival advantage (*p* = 0.06). However, in the RCRT group, CRT showed significant BCSS benefits (*p* < 0.001), which is presented in Figure 2C.

### Model recommendation behavior

We used the odds ratio (OR) obtained by multivariate logistic regression to explain the differences in characteristics between the RRT and RCRT groups generated by BITES, which is presented in Figure 2D. The presence of an OR smaller than 1 could be interpreted as a feature that might lead the model to be more likely to recommend this patient for RT treatment. We only showed the variables that guided the model to recommend RT and those with point estimates of OR value less than 1, as other variables can be considered more likely to guide the model to recommend CRT and were outside the scope of this study.

According to the OR values, patients who were males (0.36, 95% CI: 0.16–0.80), of advanced age (0.67, 95% CI: 0.61–0.72), and with tumor invasion confined to the ventricular system (0.10, 95% CI: 0.01–0.92) were more likely to be recommended for RT. Other factors that may lead to RT being recommended include being married (0.97, 95% CI: 0.43–2.14), tumor located in the temporal lobe (0.36, 95% CI: 0.09–1.28), mid (0.28, 95% CI: 0.07–1.04), across the midline (0.17, 95% CI: 0.03–1.04), tumor with larger size (1.00, 95% CI: 0.99–1.00), with metastasis (0.88, 95% CI: 0.16–4.69), having undergone biopsy (0.94, 95% CI: 0.38–2.34) and STR (0.77, 95% CI: 0.29–2.06).

### Model interpretation

**Figure 3:**
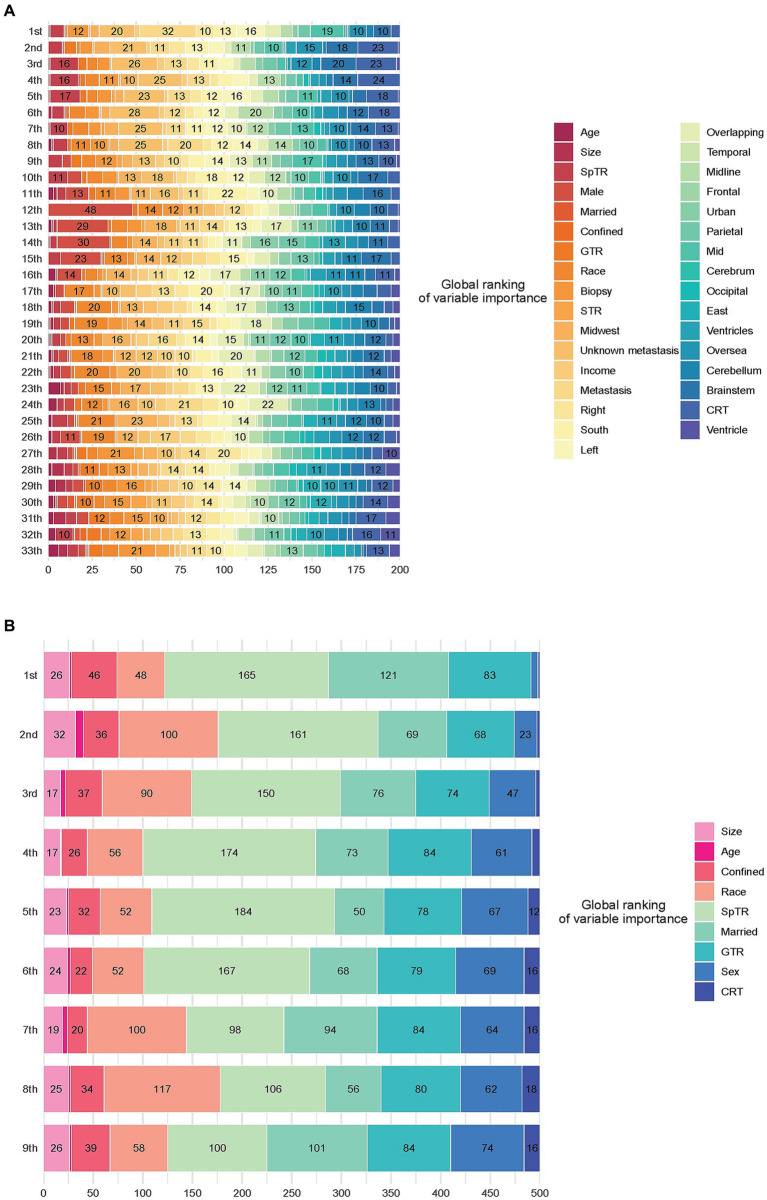
Model interpretation based on SurvSHAP(t). **(A)** Ranking the importance of all variables. **(B)** Ranking the importance of the top 8 important variables. CRT, chemoradiotherapy; STR, subtotal resection; GTR, gross total resection; SpTR, supratotal resection.

Figure 3A shows the aggregation of variable rankings over 200 observations in the treatment recommendation testing set in the BITES, and for simplicity, Figure 3B visualizes the eight most important variables sorted by aggregated SHAP values over 500 observations in the same manner. The horizontal bars represent the number of observations for which the importance of the variable, represented as a given color, was ranked as first, second, and so on. Notably, CRT in BITES was a sign of passing through different risk networks and using different baseline hazards rather than a regular variable.

Having undergone SpTR was deemed the most important prognostic factor by 165 observations, followed by being married and having undergone GTR. Voted by the majority, race was the second most important variable, marriage was the third, GTR was the fourth, and sex was the fifth.

## Discussion

The trajectory of GBM is characteristically rapid and dire, with a survival rate of about 25% at 2 years post-diagnosis and 5%–10% at 5 years (37). In 2005, a phase 3 clinical trial showed that CRT can lead to longer survival versus RT alone (10). However, despite evidence that CRT shows promise survival of 10 to 14 months (38), the high incidence of treatment-related toxicities in up to 60% of patients receiving CRT necessitates a cautious approach, especially for certain demographics like older patients with limited life expectancy (39, 40). This situation underscores the importance of patient-specific treatment selection to avoid unnecessary toxicity. For instance, identifying patients who are better suited for RT, based on individual characteristics, can significantly mitigate the risk of adverse treatment effects.

In the context of individualized treatment recommendations for GBM, methodologies such as T-learner and representation-based methods have been introduced to infer counterfactual outcomes. However, in the field of medicine, there is a lack of extensive discussion and comparison of these models, especially in their statistical approaches and ITE calculation methods (41, 42). Our study addresses this gap by evaluating the BITES model against traditional T-learner and other machine learning-based methods, revealing the former’s superior performance in the GBM domain. We believe that there are three possible reasons for the performance enhancement. First, the end-to-end training approach makes the model’s predictive ability consistent. Second, the deeper shared network, replacing a single-layer model with a multilayer perceptron, and training approach with small batches of data (43) allows for enhanced feature extraction ability. Finally, the strategy of representation balancing further reduces the selection bias (25, 26).

In the inference of ITE, the central question we focused on was: “How much will a specific patient’s BCSS outcome improve when he or she receives CRT instead of RT?.” We used HR, HR^a^, dRMST, and NNT as our core performance metrics because they directly reflect a better survival outcome in the treatment recommendation task and are statistically guaranteed by well-established statistical methods (44). Among all models, the recommendation of BITES provided the strongest protective factor. Patients whose actual treatments were consistent with model recommendations can reduce the risk of mortality by 47% and have an average of 7.92 months of additional BCSS over 5 years. After ruling out the potential of confounding and selection bias, the HR^a^ was still statistically significant. In average life gain analysis (36), 1.67 patients change treatment according to BITES recommendation can prevent an extra event in comparison with not following recommendation during the 5 years follow-up, which is estimated by the K–M method. Although CPH recommended CRT for all patients, this action resulted in a weaker protective effect than BITES, and the point estimates of all indicators of CPH were worse than those of BITES. This phenomenon suggested the significance of identifying specific populations that are more suitable for RT. The test of the K–M curves found a nonsignificant survival advantage for CRT in the RRT group (*p* = 0.06) and a significant survival advantage for CRT in the RCRT group (*p* < 0.001), indicating that the therapeutic effect of CRT is not superior to that of RT in the RRT group. The IPTW-adjusted log-rank test was not used to evaluate the treatment effect of CRT because potential differences in treatment effects may be due to patient characteristics. As the SEER database does not provide information on the response to CT, we were unable to evaluate the side effects of CRT on these patients. We recommend that patients in the RRT group be given preference for RT, as it may help to avoid the potential toxicity of CT that patients would endure.

Several studies have discussed that people of advanced age should probably not receive CRT (13, 40, 45, 46), and this research has produced more quantitative findings (OR of age: 0.67, 95% CI: 0.61–0.72). Our results also suggested that male sex (0.36, 95% CI: 0.16–0.80) and tumor invasion confined to the ventricular system (0.10, 95% CI: 0.01–0.92) were factors that led patients to be more likely recommended for RT. Metabolic and endocrine differences due to gender may be responsible for this outcome (47), which warrants further research. The model we built was highly interpretable by using SurvSHAP(t). The results reflected the prognostic predictive value of the EOR in GBM patients, which has been confirmed in several studies (48, 49). Additionally, the significance of the partial demographic and tumor-related information we identified aligns with previous clinical experience and evidence (50–53). An exception is the marital status of patients, as one prior study emphasizes that married patients might experience more beneficial treatment effects from aggressive CRT as opposed to RT alone (54).

Our model (BITES) may serve as a useful analytical tool for treatment recommendation in patients with GBM, given its evidence of the significant prognostic benefits of following the treatment recommendation, which clearly outweigh those associated with not following the recommendation. To facilitate discussion of different potential treatment options, physicians and patients need an informative tool that focuses on survival benefits. In real cases, the establishment of a treatment recommendation system based on DL models will be key to effectively conveying results and illustrating complex analyses, including prognostic prediction, treatment recommendation to patients and family members, and improving the physicians’ understanding of the treatment benefits (55, 56).

From a clinical standpoint, the findings of our study and capabilities of the BITES model present a transformative approach in the management of GBM patients. The clinical landscape of GBM is marked by the diverse responses of patients to standard treatments and severe morbidity often associated with more aggressive therapies. Our model’s insights into these dynamics are vital for advancing clinical practices in treating this challenging condition. The BITES model’s ability to accurately predict the most suitable treatment modality for each patient is a significant clinical advancement. While CRT offers a survival benefit, its effectiveness is often overshadowed by severe toxicities, which are particularly detrimental in vulnerable groups such as the elderly or those with pre-existing comorbidities (10, 57). BITES addresses this by aiding clinicians in making informed decisions, balancing the potential benefits of aggressive treatment against the associated risks, and thereby enhancing patient outcomes as much as possible.

A crucial aspect of the BITES model is its emphasis on demographic factors like age and gender, which play a significant role in determining treatment efficacy. This aligns with recent research suggesting that gender-based metabolic differences can influence treatment responses (45). By identifying patients more likely to benefit from RT over CRT, the model not only helps in reducing the incidence of treatment-related adverse effects but also promotes the principles of precision oncology and patient-centric care. This is particularly relevant in the current clinical context, where the quality of life is increasingly recognized as a critical outcome in GBM management (58). However, integrating AI-driven tools like BITES into clinical practice involves navigating complex ethical, logistical, and educational challenges. Future research should focus on validating these insights through clinical trials and exploring the model’s applicability in diverse patient cohorts. This will ensure its reliability and generalizability in practical clinical settings.

In summary, from the perspective of clinical practice, the BITES model marks a significant step forward in personalized GBM management. It promises to refine treatment decisions, reduce toxicity, and improve overall patient outcomes, heralding a new era in individualized and effective GBM therapy.

### Limitations

This study has several limitations. We have categorized the main deficiencies into two aspects: (1) the lack of information on treatment and (2) the lack of information related to tumors. Due to database limitations, we were unable to extract the information regarding therapeutic doses used by patients and the drugs used for CT, which is important (59). We also lacked some key information, such as IDH mutation and Karnofsky performance status. Meanwhile, it is also crucial to verify the reliability of the model through the implementation of a blinded prospective study so that this model can be used with confidence in clinical practice. Finally, it is difficult to avoid having the training and testing sets come from the same database, which may reduce the generalizability of the DL models. Subsequent studies should validate these models on real-world clinical data. However, we presented meaningful results based on the available variables, which could narrow the scope for subsequent research, and provided evidence for the feasibility of DL modeling for such applications.

## Conclusion

In this cohort study, several machine learning models predicted which patients with GBM would benefit from receiving CRT. Although such models are naturally opaque, some techniques can help us understand their behavior. Future studies will need to confirm the validity of these models and findings, and further analysis with more comprehensive clinical data not captured in the SEER may result in predictions that are even more accurate. BITES has the potential to distill the complex heterogeneity of real-world practice into meaningful recommendations for true precision medicine.

## Data availability statement

The original contributions presented in the study are included in the article/supplementary material, further inquiries can be directed to the corresponding authors.

## Ethics statement

Ethical approval was not required for the study involving humans in accordance with the local legislation and institutional requirements. Written informed consent to participate in this study was not required from the participants or the participants’ legal guardians/next of kin in accordance with the national legislation and the institutional requirements.

## Author contributions

EZ: Conceptualization, Formal analysis, Investigation, Project administration, Visualization, Writing – original draft. JW: Conceptualization, Formal analysis, Investigation, Visualization, Writing – original draft. WS: Data curation, Formal analysis, Investigation, Validation, Writing – original draft. QJ: Data curation, Formal analysis, Methodology, Writing – original draft. PA: Conceptualization, Data curation, Formal analysis, Methodology, Writing – original draft. DS: Conceptualization, Funding acquisition, Supervision, Validation, Writing – review & editing. ZA: Funding acquisition, Project administration, Resources, Supervision, Writing – review & editing.
